# Informed Consent in Perinatal Care: Challenges and Best Practices in Obstetric and Midwifery-Led Models

**DOI:** 10.3390/nursrep15080273

**Published:** 2025-07-29

**Authors:** Eriketi Kokkosi, Sofoklis Stavros, Efthalia Moustakli, Saraswathi Vedam, Anastasios Potiris, Despoina Mavrogianni, Nikolaos Antonakopoulos, Periklis Panagopoulos, Peter Drakakis, Kleanthi Gourounti, Maria Iliadou, Angeliki Sarella

**Affiliations:** 1 Department of Midwifery, Faculty of Health and Caring Sciences, University of West Attica, 122 43 Athens, Greece; kgourounti@uniwa.gr (K.G.); miliad@uniwa.gr (M.I.); asare@uniwa.gr (A.S.); 2 Third Department of Obstetrics and Gynecology, University General Hospital “ATTIKON”, Medical School, National and Kapodistrian University of Athens, 124 62 Athens, Greece; sfstavrou@med.uoa.gr (S.S.); apotiris@med.uoa.gr (A.P.); perpanag@med.uoa.gr (P.P.); pdrakakis@med.uoa.gr (P.D.); 3 Laboratory of Medical Genetics, Faculty of Medicine, School of Health Sciences, University of Ioannina, 451 10 Ioannina, Greece; ef.moustakli@uoi.gr; 4 Birth Place Lab, Division of Midwifery, Faculty of Medicine, University of British Columbia, Vancouver, BC V6T 1Z3, Canada; saraswathi.vedam@ubc.ca; 5 First Department of Obstetrics and Gynecology, Alexandra Hospital, Medical School, National and Kapodistrian University of Athens, 115 28 Athens, Greece; dmavrogianni@med.uoa.gr; 6 Department of Obstetrics and Gynecology, University of Patras, 265 04 Patras, Greece; antonakopoulos2002@yahoo.gr

**Keywords:** informed consent, midwifery-led care, obstetric care, perinatal period, patient autonomy, maternal rights, healthcare communication

## Abstract

**Background/Objectives:** Respectful maternity care involves privacy, dignity, and informed choice within the process of delivery as stipulated by the World Health Organization (WHO). Informed consent is a cornerstone of patient-centered care, representing not just a formal document, but an ongoing ethical and clinical process through which women are offered objective, understandable information to support autonomous, informed decision-making. **Methods:** This narrative review critically examines the literature on informed consent in maternity care, with particular attention to both obstetric-led and midwifery-led models of care. In addition to identifying institutional, cultural, and systemic obstacles to its successful implementation, the review examines the definition and application of informed consent in perinatal settings and evaluates its effects on women’s autonomy and satisfaction with care. **Results:** Important conclusions emphasize that improving women’s experiences and minimizing needless interventions require active decision-making participation, a positive provider–patient relationship, and ongoing support from medical professionals. However, significant gaps persist between legal mandates and actual practice due to provider attitudes, systemic constraints, and sociocultural influences. Women’s experiences of consent can be more effectively understood through the use of instruments such as the Mothers’ Respect (MOR) Index and the Mothers’ Autonomy in Decision Making (MADM) Scale. **Conclusions:** To promote genuinely informed and considerate maternity care, this review emphasizes the necessity of legislative reform and improved provider education in order to close the gap between policy and practice.

## 1. Introduction

Informed consent is the cornerstone of ethical, patient-centered, and human rights-based healthcare, particularly in midwifery and obstetric care [1]. Pregnant women should be thoroughly informed about their options, including potential dangers, advantages, and alternatives. This goes beyond simply giving them information. In person-centered care, even if patients do not actively participate, decision-making is essential and linked to better healthcare outcomes [2]. According to research, women’s decisions during pregnancy and childbirth are greatly influenced by the kind and caliber of information they are given [3,4]. International medical and bioethical accords generally acknowledge the significance of informed consent in healthcare. The need for informed consent in healthcare is solidly based on foundational principles like the Nuremberg Code (1947), Declaration of Helsinki (1964), and Council of Europe’s Convention on Human Rights and Biomedicine (1997), and it is further reaffirmed by global bodies including the World Health Organization (WHO), UNESCO, Council of International Organizations of Medical Sciences (CIOMS), International Federation of Gynecology and Obstetrics (FIGO), and the International Confederation of Midwives (ICM) [5,6,7].

Improved perinatal health as a result of advances in medical technology have also been accompanied by complex decisions and ethical issues and added demands for informed and shared decision-making. At the same time, greater education and awareness among women have raised their expectations for respectful and individualized care [8]. At the same time, women’s increasing autonomy and education have raised hopes for shared decision-making, respect, and individualized care [9,10]. Various studies have noted that a woman’s birth experience is influenced as much by clinical results as by how much control she exercises, how well she is communicated with by providers, and to what extent she is involved in decisions [3,4,11,12].

According to Vedam and colleagues, women report having limited control over decisions about their prenatal care, despite these ethical and legal frameworks [12,13,14]. The degree of medical staff assistance, the caliber of patient–provider contacts, and decision-making involvement are three important aspects that impact delivery experiences [11]. While there are instruments to evaluate shared decision-making (SDM) in general healthcare (National Institute for Health and Care Excellence, 2014) and prenatal care, the measurement of informed consent in maternity care is not commonly practiced [15]. Their ability to adequately reflect the experiences of expecting women is limited because healthcare professionals rather than service consumers developed the most validated SDM measures.

Patient perspectives improve the quality and relevance of biomedical research, according to Caron-Flinterman and colleagues [16]. In high- and middle-income nations, there is no globally accepted scale for evaluating respectful and informed consent procedures in maternity care. Although various instruments have been created, including the Mothers on Respect (MOR) Index and the Mother’s Autonomy in Decision Making (MADM) Scale, no official international organization has developed a standardized and internationally accepted tool to measure and monitor informed consent practices over time in perinatal care. The measurement of informed consent in perinatal care is in its early stages [17].

The purpose of this narrative review is to close this gap by critically analyzing maternity services’ informed consent procedures, with an emphasis on both obstetric-led and midwifery-led care models. To better align clinical practice with patient-centered care and existing policy frameworks, this review examines the definition and application of informed consent in perinatal care within obstetric-led and midwifery-led approaches. It identifies key obstacles to effective implementation and evaluates the impact of current practices on women’s psychological well-being and decision-making autonomy. Finally, it investigates evidence-based strategies to improve informed consent processes in maternity care.

This review was guided by research questions concerning the definition and application of informed consent in obstetric-led and midwifery-led models of perinatal care, the primary obstacles to obtaining effective informed consent in these settings, the impact of current practices on women’s autonomy and satisfaction with maternity care, and the approaches proposed to improve informed consent in maternity services. By addressing these questions, this review seeks to provide a comprehensive synthesis of existing evidence, highlight gaps in current practice, and offer insights to inform future improvements in maternity care.

## 2. Materials and Methods

### 2.1. Information Sources

This comprehensive research aimed to investigate the efficacy, challenges, and application of informed consent in care led by midwives and obstetricians. A thorough narrative literature review was conducted using academic databases, including PubMed/Medline, Scopus, and CINAHL, along with additional relevant sources identified through reference list screening (snowballing method).

### 2.2. Search Strategy

The search strategy employed Boolean operators (AND, OR) to refine the results using terms such as “informed consent,” “midwifery-led care,” “obstetric care,” “perinatal care,” and “patient autonomy.” Searches were conducted between January and March 2025. The search equations for each database were as follows:

- PubMed:

(“Informed Consent”[MeSH Terms] OR “informed consent”[Title/Abstract]) AND (“Midwifery”[MeSH Terms] OR “midwifery-led care”[Title/Abstract]) AND (“Obstetrics”[MeSH Terms] OR “obstetric care”[Title/Abstract]) AND (“Perinatal Care”[MeSH Terms] OR “maternity care”[Title/Abstract]) AND (“Patient Autonomy”[MeSH Terms] OR “decision-making autonomy”[Title/Abstract]).

- Scopus:

TITLE-ABS-KEY(“informed consent”) AND TITLE-ABS-KEY(“midwifery-led care” OR “midwifery”) AND TITLE-ABS-KEY(“obstetric care” OR “obstetrics”) AND TITLE-ABS-KEY(“perinatal care” OR “maternity care”) AND TITLE-ABS-KEY(“patient autonomy” OR “decision-making”).

- CINAHL:

(MH “Informed Consent”) AND (MH “Midwifery”) AND (MH “Obstetrics”) AND (MH “Perinatal Care” OR “Maternity Care”) AND (MH “Patient Autonomy” OR “Decision Making”).

### 2.3. Inclusion and Exclusion Criteria

The inclusion criteria encompassed peer-reviewed articles published between 2000 and 2025 that specifically examined informed consent as a distinct construct in perinatal care, including its definition, application, barriers to implementation, and impact on women’s autonomy and psychological well-being. Studies focusing solely on broader aspects of decision-making participation or quality of care without direct reference to informed consent were excluded.

Only articles using validated tools—such as the Mother’s Autonomy in Decision Making (MADM) Scale or Mothers on Respect (MOR) Index, or those offering relevant qualitative or quantitative insights—were included. Studies published in English were included, while studies in other languages were excluded due to resource limitations, which may limit the representativeness of findings. Studies that did not directly address informed consent within obstetric or midwifery contexts were excluded. Although not all included studies utilized standardized measures of informed consent, they were retained due to their relevance in exploring consent processes, communication dynamics, and women’s autonomy within perinatal care.

### 2.4. PICO-Based Research Question and Eligibility Criteria

In pregnant women receiving maternity care (P), does the implementation of structured informed consent practices (I), compared to routine care without standardized consent protocols (C), affect informed consent quality (e.g., comprehension, voluntariness) and subsequently influence patient autonomy, satisfaction, and psychological well-being (O)? Table 1 summarizes the key elements of the PICO framework and Table 2 the applied selection criteria.

### 2.5. Study Selection Process

**Figure 1:**
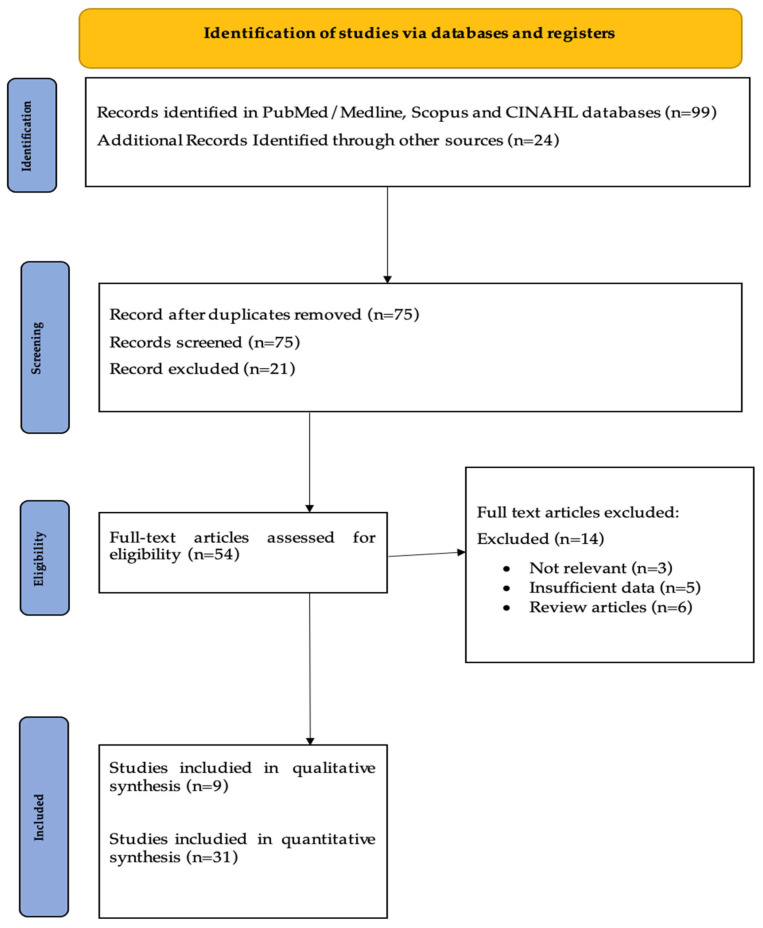
Literature search and study selection process.

An initial search yielded 99 records. After removing 24 duplicates, 75 articles were screened by title and abstract, resulting in the exclusion of 21 studies. The remaining 54 full-text articles were assessed for eligibility based on predefined criteria, leading to the exclusion of 30 studies. Ultimately, 24 studies were included in the final review. Study selection was conducted independently by two authors (E.K. and S.S.), with any disagreements resolved by a third author (A.S). Additionally, reference lists of included articles were screened using the snowball method to identify further relevant studies. The literature search and study selection process are summarized in Figure 1.

While certain studies exhibited limitations such as small sample sizes and limited generalizability, the overall methodological quality was assessed as moderate to high. The variation in contexts and methodologies was reflected in the range of key themes and conclusions reported.

### 2.6. Data Extraction and Analysis

Data extraction focused on study design, sample size, informed consent assessment methods and outcomes related to informed consent, patient autonomy and decision-making.

### 2.7. Thematic Analysis

A thematic analysis was conducted to explore the quality of informed consent, barriers to its implementation, and facilitators of the process. The research team employed an inductive coding approach, consisting of open and axial coding, to identify recurrent patterns. Codes were manually developed and grouped into overarching themes reflecting barriers, facilitators, and experiences of informed consent. No software (e.g., NVivo, etc.) was used; coding was conducted using spreadsheets and verified independently by two reviewers (E.K. and S.S.). Themes were reviewed and refined iteratively until consensus was reached. All extracted data were reviewed collaboratively by the core research team.

## 3. Results

This research sought to examine how informed consent processes and communication quality influence women’s autonomy and perinatal decision-making. Women’s autonomy is conceived as their capacity to exercise informed decisions regarding perinatal care and the place where they deliver [18]. However, their autonomy is commonly undermined by over-medicalization and disempowering approaches to care that foster dependency [19]. Obstetric care performance is primarily measured by health outcomes, such as maternal and child mortality and morbidity in both the short and long term, with variations observed globally [12,20]. This review included empirical studies as well as systematic reviews to comprehensively capture existing evidence on informed consent practices and their impact. The methodological quality of included studies was appraised using validated tools appropriate to each study design, such as the CASP checklist for qualitative research, the Newcastle–Ottawa Scale for quantitative studies, and the AMSTAR 2 tool for systematic reviews. These assessments guided the synthesis by weighting findings based on study rigor.

Decision-making is shown to be associated with reduced unneeded obstetrical interventions [11]. Disempowerment and disrespect of women during perinatal care makes women frequently feel infantilized and frightened by caregivers, and like they have little power over the decision-making process [21]. The selected interventions do not suit their preferences and inadequate information regarding alternative options was provided. Better information about the need for and results of specific procedures was requested by women [22]. Poor communication and loss of autonomy were shown to be types of abuse in perinatal care, leading to the conclusion that enhancing communication between women and care providers could help resolve these concerns [23]. Additional research has corroborated these findings, showing that interventions commonly imposed without meaningful informed consent ranged from coercion to the administration of treatments against women’s expressed preferences or without prior authorization [22,24]. These unpleasant experiences have been connected to postpartum depression, PTSD, and fear of giving birth in subsequent pregnancies [21]. PTSD is a result of unpleasant childbirth experiences, frequently brought on by a lack of participation in decision-making and a perception of insufficient care [25].

One core research question focused on whether communication and informed consent influence women’s experiences during childbirth. Some studies included in this review observed that women who received continuous care and informed consent throughout the perinatal period reported more satisfying experiences and, in certain cases, fewer interventions compared to other models of care [2]. Women’s active participation in their own care is related to more satisfying health professional experiences, lower costs, better health results, and greater satisfaction with their care [2]. Again, however, it is essential to say that these results do show an association rather than causation between informed consent and all of these results [1]. There is presently insufficient data from which to make informed conclusions about how informed consent makes a difference to birth outcomes and little evidence about its effect in maternity settings [26]. These shortcomings demonstrate a need for more studies that are able to test more rigorously the association of informed consent and outcomes in relation to birth [26].

Another research question addressed the role of provider behavior in shaping informed consent practices. Research indicates that care provider attitudes and behaviors have a more significant impact on women’s experiences than factors such as socioeconomic status, race, physical environment, and medical interventions [27]. Midwives are more engaged with shared decision-making than doctors in high-income countries. Pre-service and in-service training gaps for informed consent, as well as for patients’ rights, are larger among lower-tier providers in Low–Middle Income Countries (LMICs) [28]. It is important to note that while these studies highlight valuable insights, the varying cultural and healthcare contexts in which they were conducted may affect the generalizability of these findings [28,29]. The reliability and validity of tools like the MOR Index and MADM Scale in these diverse settings should be further assessed to ensure that they accurately capture women’s experiences of autonomy in perinatal care.

The instruments used to assess autonomy in perinatal care were also investigated. Heaman and associates demanded in a related study that trustworthy and validated research instruments be created to gauge women’s experiences of violence during childbirth [15]. The MOR Index and MADM Scale are two instruments that quantify patient experiences with informed consent by gauging how women view their role in decision-making. According to studies employing these instruments, a large number of women experience pressure to participate in interventions before fully comprehending or consenting to them [13,14,28]. These tools have shown that many women feel pressured into procedures they do not fully understand or have not consented to. The use of standardized tools is essential for identifying gaps in consent practices and supporting system-wide improvements in maternity care [13,14,28]. Many women in maternity care say they were given unfair or incomplete information when choosing where to give birth [15,18]. It is important to provide information that is tailored to individual requirements and supported by the latest scientific evidence, as Dektar and colleagues found that women were often unaware that they could give birth outside of a hospital [18]. Autonomy and decision-making depend on choice, which is informed by adequate knowledge [23].

Informed consent is commonly implemented, even though it is recognized by international criteria established by the WHO and ICM. Despite the existence of laws, their application varies substantially according to the healthcare setting [10,30]. Time constraints and provider attitudes hinder effective informed consent. Midwives and obstetricians often view the process as time-consuming, leading to rushed or inadequate discussions [14].

Furthermore, the paternalistic model of medical decision-making, which traditionally involved healthcare practitioners making decisions for patients, still limits women’s agency in prenatal care decisions despite the increasing shift toward patient-centered care [31]. Sociocultural issues such as language barriers and cultural norms further hinder informed decision-making, especially for women from underprivileged areas who face systemic biases and power imbalances [10]. Moreover, consent procedures should also be adapted based on women’s literacy levels. These strategies involved pictorial supports, verbal explanation via local languages, and culturally appropriate narrative strategies for enabling understanding and agency [10,28,29]. Studies recommend using culturally sensitive consent approaches with visual aids, community stories, and peer counselor or doula support in order to improve understanding and agency [10,14,28,29,32].

In addition, whether or not informed consent practices influence clinical outcomes remains to be addressed through the research. Although informed and respectful care has been postulated to be associated with enhanced patient satisfaction as well as trust in practitioners and adherence to treatment plans, and while evidence supporting these associations exist, their causal nature is under-explored. There is insufficient strong evidence in the existing literature regarding whether informed consent influenced maternal and neonatal outcomes, indicating a compelling need for additional studies in LMICs [1,26,32]. However, the variation in the application of these strategies across different healthcare systems and settings may impact their effectiveness [16,26,32]. Future research should explore how these strategies can be tailored to address the specific needs and challenges of different populations, especially in low- and middle-income countries [32]. Table 3 summarizes the studies included in the review, alongside their key findings and methodological characteristics.

### Critical Appraisal of Included Studies

The studies covered in this review show various methodological strengths and weaknesses that should be taken into account when evaluating the results. Systematic reviews, including those conducted by Ayudiah et al. (2024) [1], Barry and Edgman-Levitan (2012) [2], and Bohren et al. (2015) [32], offer thorough summaries of available evidence and important insights; however, some are constrained by geographic concentration or scope, which could influence their generalizability. For example, Ayudiah et al. (2024) [1] concentrate on midwifery methods in Pakistan, potentially restricting the relevance of their results to different situations. Extensive quantitative research, such as that by Vedam et al. (2019) [12] and Lukasse et al. (2015) [21], provides strong data and highlights significant risk factors like racial inequalities in mistreatment, but their cross-sectional design limits causal conclusions, and the focus on U.S. research may restrict wider applicability. Qualitative and interview-driven research provides a deep contextual comprehension of women’s experiences with informed consent and decision-making, improving awareness of psychosocial and cultural influences; nevertheless, these studies usually involve limited samples and can encounter issues with generalizability. Utilizing validated tools such as the MADM Scale and MOR Index across various studies enhances the credibility of autonomy-related results, but incorporating studies lacking standardized instruments adds variability. Certain studies underscore significant systemic problems, such as the restricted legal responsibility of providers and their grasp of informed consent (e.g., Kruske et al., 2013 [27]). Although the variety of approaches adds to the body of information, it also introduces variation in population, study design, and measurement techniques. These limitations are recognized and taken into account when interpreting the findings and suggesting best practices for informed consent during pregnancy.

## 4. Discussion

This review highlights the centrality of informed consent in maternity care, while also demonstrating that its implementation varies significantly across different healthcare systems and cultural contexts. While formal regulations and policies exist, their practical application frequently falls short of ensuring respectful and informed decision-making. Important conclusions show that several factors can prevent appropriate consent, such as sociocultural impacts, enduring medical paternalism, and provider time limits [33,34]. In low-resource settings, linguistic barriers, systemic inequalities, and insufficient educational opportunities further compound these challenges. Comparable initiatives remain scarce or insufficiently studied in low- and middle-income countries, while high-income nations increasingly implement formal tools and training programs to strengthen the consent process. The research underscores the pressing need for scalable and contextually nuanced strategies to promote patient autonomy and improve informed consent practices on a global scale.

Research indicates that obstetricians and midwives often perceive the informed consent process as time-consuming, leading to rushed or incomplete discussions [12]. Historically, medical decision-making was dominated by a paternalistic model, where healthcare providers made decisions on behalf of patients. Although patient-centered care is gaining traction, remnants of this approach persist, limiting women’s agency in perinatal care decisions [35]. Additionally, cultural expectations, language barriers, and systemic biases disproportionately affect marginalized communities, further hindering informed decision-making [12].

The extent and impact of these barriers can vary across different healthcare settings, cultural contexts, and healthcare systems. For instance, while many studies in high-income countries report time constraints and medical paternalism as significant barriers, research in low- and middle-income countries often highlights the challenges posed by cultural influences, power imbalances, and limited access to education and healthcare resources. These variations underscore the need for context-specific strategies to address these barriers [36,37]. Research designs, including mixed-methods, qualitative, and quantitative approaches, were used in the included studies. Both large-scale quantitative research with hundreds of participants and smaller qualitative studies with less than 20 participants had sample sizes that varied widely. These investigations were conducted across multiple geographical regions and diverse healthcare settings. While certain studies exhibited limitations such as small sample sizes and limited generalizability, the overall methodological quality was assessed as moderate to high. The variation in contexts and methodologies was reflected in the range of key themes and conclusions reported. To make practical implementation feasible, we recommend a tiered approach that is sensitive to varying levels of resources. For high-resource settings, this includes standardized training on communication and ethics, digital consent tools, and audit systems. For low- and middle- resource settings, priority can be given to streamlined consent forms, culturally appropriate oral explanation, and task-shifting to trained community health workers [38].

Variability is also driven largely by healthcare infrastructure, legal protections, and cultural norms. For example, patient decision aids may be possible for technology-accessed tertiary hospitals but not for primary care facilities with limited electricity in rural areas. Cultural factors, such as family decision-making roles and respect for authority figures, can challenge autonomy-based consent models, especially in regions like Southeast Asia and Sub-Saharan Africa [39].

Women possess valuable knowledge that can contribute to the improvement of healthcare systems, policies, and their treatment [40]. The quality and safety of healthcare services could be greatly improved by making efficient use of this important resource [41]. Regarding maternal requests for cesarean sections, respect for patient autonomy is particularly crucial. To ensure autonomy, the mother’s informed consent must be obtained [42,43]. However, a growing number of parents, obstetricians, and midwives believe that more research on this subject is neither suitable nor practicable [44].

Although some studies discussed how information was communicated to women, few specifically addressed adapting consent procedures to different literacy levels. This is an important aspect of informed consent, and future research should examine the effectiveness of communication strategies tailored to women’s literacy and education levels to ensure that all patients can make truly informed decisions [45]. Future research should evaluate visual, verbal, or culturally tailored strategies to ensure understanding across all demographics [45]. Incorporating findings from high-quality observational studies into clinical guidelines could further improve consent practices [46].

Implementing structured programs such as Canada’s Maternity Care Education Program and Respectful Maternity Care Training in several low- and middle-income countries has demonstrated improvements in consent quality. Practical examples include standardized procedures guided by the UK’s NICE guidelines, American community health initiatives offer multilingual and culturally appropriate information, which enhances patient confidence through individualized decision support [27,47]. Health systems must invest in scalable approaches tailored to their unique challenges, from infrastructure limitations to provider-to-patient ratios and prevailing cultural norms. Policymakers should prioritize institutional changes that integrate ethical training, structural support, and performance audits within maternity care delivery.

This review also employed MADM and MOR Index instruments to evaluate decision-making and respect. While valuable, these tools may not fully capture women’s experiences in diverse social contexts, underscoring the need for further validation studies across populations. As a narrative review, our findings are subject to limitations including potential publication bias and heterogeneity in study designs and populations, restricting broad generalizability.

Ultimately, equitable and effective informed consent requires embracing context-sensitive, scalable solutions. Future research should focus on testing culturally and linguistically responsive tools, assessing the impact of provider training, exploring institutional accountability mechanisms, and prioritizing studies among underrepresented groups, especially in low- and middle-income countries [24,48,49,50,51]. Beyond legislative mandates, women’s autonomy must be integrated as a standard element of clinical practice worldwide. Most current evidence arises from Western or high-income settings, limiting its applicability elsewhere. Additional research is needed to understand how informed consent is practiced across diverse healthcare structures and cultures.

Moreover, future investigations could assess the real-world effectiveness of various strategies in different resource environments and population groups. Although some data exist on the relationship between healthcare provider training and informed consent efficacy in high-income countries, such evidence remains scarce in low- and middle-income settings. Understanding how professional education and healthcare infrastructure impact implementation is crucial. Given the wide variation in healthcare systems—from well-resourced institutions to resource-limited facilities—tailored approaches are essential to optimize informed consent practices and promote patient autonomy effectively.

Maternity care based on equitable and effective informed consent during the perinatal period is not a clinical necessity but an ethical imperative that health professionals (obstetricians and midwives) need to implement. As health systems all over the world continue to develop rapidly, informed consent practices must move beyond formality to embody genuine respect for women’s autonomy, cultural context, and decision-making dimensions. Health providers’ education regarding communication tools needs to be prioritized so barriers can be avoided effectively. Educational strategies need to focus on culturally validating interventions, strengthening institutional accountability, and boosting the voices of women.

## 5. Limitations

There was no formal danger of bias or use of quality grading systems because this was a narrative review. The results of several research works were combined to identify recurring themes and trends, which allowed for the drawing of more general conclusions regarding the condition of informed consent in modern maternity care. The exclusion of pertinent studies published in other languages may also have resulted from the linguistic restrictions to English and Spanish.

## 6. Conclusions

Informed consent is a crucial right in maternity care, established in legal and ethical norms worldwide. Nonetheless, this review uncovered persistent difficulties in its practical implementation across various nations and care systems. Frequent obstacles include insufficient training for providers, inconsistency in consent procedures, restricted communication between patients and providers, and cultural or systemic elements that limit women’s independence. Research also uncovered variations in the quality of consent tied to the availability of resources and healthcare environments, highlighting the necessity for contextually appropriate solutions.

Systemic changes are necessary for effective improvements, including improved training for maternity care providers on informed consent principles, the creation and application of standardized consent procedures, and the promotion of patient-centered communication that actually supports women’s choices. Healthcare systems may eliminate inequities, provide autonomous and respectful maternity care, and ultimately enhance maternal and newborn outcomes by tackling these challenges. This review emphasizes that although a worldwide framework is crucial, adapting strategies to local contexts is vital for bridging current gaps and promoting fair maternity care.

## Figures and Tables

**Table 1:** PICO framework and inclusion/exclusion criteria.

PICO Element	Inclusion Criteria	Exclusion Criteria
Population (P)	Pregnant women in perinatal care settings (prenatal, intrapartum, postpartum); all ages and parities. Studies with mixed populations included only if data specific to pregnant women can be extracted.	Non-pregnant individuals; non-human studies; pediatric or non-maternal care; studies where pregnant women’s data are not separable.
Intervention (I)	Studies addressing informed consent in maternity care using tools (e.g., MADM, MOR); shared decision-making.	No relevance to informed consent; general ethics without maternity focus.
Comparator (C)	Usual care or non-structured consent protocols as baseline.	No comparator; non-empirical opinion articles.
Outcomes (O)	Autonomy, satisfaction, PTSD, mistreatment, frequency of interventions.	No patient-centered outcomes; purely technical/clinical results.

**Table 2:** This table summarizes the key study selection criteria applied in the review.

Criteria	Inclusion	Exclusion
Study	Qualitative, quantitative (cross-sectional, cohort), systematic reviews	Case reports, editorials, abstracts without full text
Language	English	Other languages not translated
Data Range	2000–2025	Outside 2000–2025 range

**Table 3:** Studies included in the review with their main outcomes and study design.

Study	Country	Study Design	Sample	Outcomes	Key Findings
Ayudiah, F. et al. (2024) [1]	Pakistan	Systematic Review	-	Investigates the implementation of informed consent in independent midwifery. Focuses on communication processes and legal obligations. Examines Midwife Nela’s practice.	Suggestions for improving health policies and practices. Strengthen legal protections for both patients and healthcare providers. Specifically targets the midwifery sector.
Barry, M. J. and Edgman-Levitan, S. (2012) [2]	USA	Perspective	-	Active engagement of patients when making fateful healthcare decisions. Patients face diverging medical options with significant consequences.	Patients and families are often excluded from important discussions. They feel uninformed about how their health problems are manage. Difficulty navigating complex diagnostic and treatment options.
Thomson, G. et al. (2013) [10]	USA	Semi-Structured Interviews	86	Antenatal care attendance. Frequency of antenatal appointments. Location of antenatal care. Provision of risk information.	Offers a new paradigm in public health. The level of service is proportionate to need.
Vedam, S. et al. (2019) [12]	USA	Quantitative Research	2700	Women of color experienced consistently higher rates of mistreatment. This was true even when interactions between race and other maternal characteristics were examined.	Mistreatment is more frequent among women of color, those giving birth in hospitals, and those facing social, economic, or health challenges. Mistreatment is exacerbated by unexpected obstetric interventions and patient–provider disagreements.
Vedam, S. et al. (2017) [14]	USA	Quantitative Research	2514	A reliable tool for assessing decision-making experiences during maternity care. The scale was developed and content validated by community members. Includes women from diverse and vulnerable populations in BC.	Reliably assesses interactions with maternity providers. Evaluates a person’s ability to lead decision-making throughout maternity care.
Hodnett, E. D. et al. (2012) [11]	USA	Systematic Review	10,684	Established for pregnant women who prefer minimal or no medical intervention. Provide care for women who require or prefer less medical involvement during pregnancy.	No significant impact on serious perinatal or maternal morbidity/mortality. Alternative versus conventional institutional settings for birth.
Heaman, MI. et al. (2014) [15]	USA		80	Confirmed that women’s ratings of prenatal care quality remained consistent. Ratings did not change after giving birth or between the early postpartum period and 4 to 6 weeks postpartum.	Valid and reliable tool for assessing quality of care. Useful in future research as an outcome measure. Can compare quality of care across geographic regions, populations, and service delivery models. Evaluates the relationship between quality of care and maternal and infant health outcomes.
Caron-Flinterman, JF, Broerse, JEW, Bunders, JFG. (2005) [16]	USA	Quantitative Research	60	Twenty-one cases of patient participation in biomedical research identified. Concrete use of patients’ experiential knowledge traced in 9 of these cases.	Patients’ experiential knowledge can contribute to biomedical research. When translated into explicit demands, ideas, or judgments, it enhances the relevance and quality of research.
Green, JM, Baston, HA (2003) [18]	USA	Quantitative Research	1146	Feeling in control of one’s behavior and during contractions was linked to: 🗸 Aspects of pain and pain relief. 🗸 Antenatal expectations of control. Worry about labor pain was a significant antenatal predictor, especially for primiparas (first-time mothers).	All three types of control (internal, external, and control during contractions) were important to women and contributed to psychological outcomes. Internal and external control were predicted by different groups of variables. Caregivers have the potential to make a significant difference to a woman’s experience of childbirth.
Warren, C. et al. (2013) [19]	Nairobi	Quantitative Research	12	Conduct implementation research to design, test, and evaluate an approach. Significantly reduce disrespectful and abusive (D&A) care of women during labor and delivery in facilities.	Determine the manifestations, types, and prevalence of D&A care in childbirth. Develop and validate tools for assessing D&A. Identify and explore the potential drivers of D&A. Design, implement, monitor, and evaluate the impact of interventions to reduce D&A. Document and assess the dynamics of implementing interventions and generate lessons for large-scale replication
Nijagal MA. et al. A. et al. (2018) [20]	USA	Systematic Review	-	Clinical Outcomes: Maternal and neonatal mortality and morbidity; Stillbirth; Preterm birth; Birth injury. Patient-Reported Outcome Measures (PROMs): Health-related quality of life (HRQoL); Mental health; Mother–infant bonding; Confidence and success with breastfeeding; Incontinence; Satisfaction with care and birth experience.	Care for women and infants. Pregnancy and postpartum period evaluation.
Lukasse, M. et al. (2015) [21]	USA	Quantitative Research	6923	One in five pregnant women attending routine antenatal care reported some lifetime abuse in healthcare. Prevalence varied significantly between countries. Characteristics of Women Reporting Abuse: 🗸 Higher prevalence of other forms of abuse. 🗸 Economic hardship and negative life events. 🗸 Lack of social support. Symptoms of post-traumatic stress and depression.	Abuse in healthcare is common among women attending routine antenatal care. Women experiencing severe current abuse in healthcare are more likely to: 🗸 Have a fear of childbirth. 🗸 Wish for a cesarean section.
O’Connor AM. et al. (2009) [22]	USA	Systematic Review	-	Make decisions explicit. Provide information about options and associated benefits/harms. Help clarify alignment between decisions and personal values.	More knowledgeable. Better informed. Clearer about their values. More active in decision-making. Have more accurate risk perceptions. Growing evidence: Decision aids may improve value-congruent choices.
Baker, SR. (2015) [23]	USA	Interviews	24	There is a recognized need for a multidimensional or holistic approach to maternity care. The approach should include both psychological and physical aspects. Goal: To optimize women’s experiences in both the intra- and postpartum periods. The relationship between women and maternity care staff is key to this approach.	Maternity care staff must recognize women’s psychological and emotional needs during childbirth. Care providers’ actions can significantly affect women’s experiences. The issues are explored within the broader debate on authority and power in the medical relationship. The discussion is framed from a feminist viewpoint.
Jou, J. et al. (2015) [24]	USA	Quantitative Analysis	2400	To determine if patient-perceived pressure from clinicians for labor induction or cesarean delivery is significantly associated with undergoing these procedures. Examining the relationship between perceived clinician pressure and the likelihood of having labor induction or cesarean delivery.	Patient-perceived pressure from clinicians significantly predicts labor induction and cesarean delivery. Recommended efforts: 🗸 Reduce provider-patient miscommunication. Minimize potentially unnecessary procedures.
Creedy, D. et al. (2000) [25]	USA	Quantitative Research	499	Little is known about the relationship between women’s birthing experiences and the development of trauma symptoms. Exploring how childbirth experiences may contribute to trauma symptom development.	Posttraumatic stress disorder (PTSD) after childbirth is a poorly recognized phenomenon. Risk factors: Women who experienced: 🗸 A high level of obstetric intervention; 🗸 Dissatisfaction with their intrapartum care. These women were more likely to develop trauma symptoms compared to those who received a high level of obstetric intervention or perceived their care to be inadequate.
Kruske, S. et al. (2013) [27]	USA	Quantitative Research	336	Little is known about care providers’ views on the situation. There is limited understanding of their legal accountability for outcomes during pregnancy and birth.	Maternity care professionals inconsistently supported women’s rights to autonomous decision-making during pregnancy and birth. This issue is further complicated by care providers’ poor understanding of their legal accountability for outcomes experienced in pregnancy and birth.
Declercq, E. R. et al. (2013) [28]	USA	Research Survey	2400		Describes experiences from before pregnancy through the early postpartum period. Reported in Listening to Mothers III: Pregnancy and Birth.
Shay, LA, Lafata, JE. (2014) [29]	USA	Systematic Review	-	There is broad support for shared decision-making (SDM). Empirical evidence on its effectiveness in improving patient outcomes has not been systematically reviewed.	When patients perceive shared decision-making (SDM), it tends to result in improved affective–cognitive outcomes. There is a lack of evidence linking empirical measures of SDM with patient behavioral and health outcomes.
Baker, S. and Precilla, Y. (2005) [23]	USA	Cross-Sectional Survey	1672	Abuse of human rights during childbirth is documented in low-, middle-, and high-resource countries.	A reliable, patient-informed quality and safety indicator. Application: Can be applied across jurisdictions. Assesses the nature of provider–patient relationships and access to person-centered maternity care.
Alvarez, M, Hotton, EJ, Harding, S, Ives, J, Crofts, JF, Wade, J. (2023) [26]	USA	Interviews	51	Recruitment to intrapartum research is challenging. Women must understand unfamiliar terminology. They are asked to assess potential harm versus benefit to themselves and their baby.	Women prefer information provision and research discussion during the antenatal period. Recruitment processes in intrapartum studies still vary. There is inconsistency in how recruitment is offered to women during labor.
O’Neill, O. (2003) [30]	USA	Systematic Review	-	Considered valuable because it supports individual autonomy. There are many different views on individual autonomy. The ethical significance of autonomy varies across these conceptions.	To limit deception and coercion. Patients should have control over the amount of information they receive. Patients should have the opportunity to withdraw consent already given.
Kruk, M. et al. (2014) [31]	Tanzania	Quantitative	1779	Highlighted humiliating treatment of women during labor and delivery. Lack of reliable estimates	Health system crisis. Urgent solutions needed: 🗸 Ensure women’s right to dignity in healthcare. 🗸 Improve the effective utilization of facilities for childbirth. Goal: To reduce maternal mortality.
Bohren, M. A. et al. (2015) [32]	USA	Systematic Review	-	There is increasing awareness of neglectful, abusive, and disrespectful treatment of women during childbirth in health facilities. Lack of consensus.	Systematic review. Mistreatment levels: 🗸 Occurs at the level of interaction between the woman and provider. Results from systemic failures at the health facility and health system levels.

## Data Availability

Data sharing is not applicable. No new data were created or analyzed in this study.

## Public Involvement Statement

No public involvement in any aspect of this research.

## Guidelines and Standards Statement

This manuscript is a narrative review and does not entirely follow a specific standard.
